# Sex differences in traumatic brain injury and later‐life MRI regional brain volume associations in the Framingham Heart Study

**DOI:** 10.1002/alz70856_100027

**Published:** 2025-12-24

**Authors:** Chad William Farris, Shruti Durape, Rebecca Burton, Kurtis Chien‐Young, Eden Price, Prajakta S. Joshi, Yulin Liu, Ting Fang Alvin Ang, Ashita S. Gurnani, Douglas I Katz, Yorghos Tripodis, Michael L Alosco, Kristen Dams‐O'Connor, Rhoda Au, Jesse Mez

**Affiliations:** ^1^ Boston University Alzheimer's Disease Research Center, Boston, MA, USA; ^2^ Boston University Chobanian & Avedisian School of Medicine, Boston, MA, USA; ^3^ Boston Medical Center, Boston, MA, USA; ^4^ Framingham Heart Study, Boston University Chobanian & Avedisian School of Medicine, Boston, MA, USA; ^5^ Boston University Chronic Traumatic Encephalopathy Center, Boston, MA, USA; ^6^ Boston University Henry M. Goldman School of Dental Medicine, Boston, MA, USA; ^7^ Icahn School of Medicine at Mount Sinai, New York, NY, USA; ^8^ Boston University School of Public Health, Boston, MA, USA

## Abstract

**Background:**

Reductions in MRI brain volumes after traumatic brain injury (TBI) have been widely reported, but study samples have been highly selected, brain regions involved vary amongst studies, and sex differences have not been well studied.

**Method:**

In this cross‐sectional, community‐based analysis, Framingham Heart Study Generation‐2 cohort participants with a TBI history prior to a 1.5T MRI performed between 2005‐2008 were matched (1:4) on birth year and sex with controls without TBI. Information on TBIs was collected across the lifespan by comprehensive review of medical records, FHS health history updates and exams and self‐report. TBI occurrence and severity were operationalized using modified American Congress of Rehabilitation Medicine and Veterans Affairs/Department of Defense criteria respectively. MRI volumetric analysis using custom in‐house software produced volume measurements for the following supratentorial structures: total intracranial volume (TCV), total brain, total gray matter, total white matter, lateral ventricles, hippocampi, and gray and white matter volume in the frontal, parietal, occipital, and temporal lobes. We assessed TBI group differences in volumes, adjusting for TCV, sex, education, MRI age, APOE e4 status, and TBI‐MRI time differences. Sex‐stratified analyses were also performed. Analyses were false discovery rate corrected for multiple comparisons.

**Result:**

Among 795 participants (mean age at MRI: 67.78.6; 59% female), there were 159 participants with TBI (141 mild, 18 moderate‐severe) and 636 participants without TBI. Mean time from TBI to MRI was 11.68.9 years. Participants with TBI had significantly smaller hippocampal volumes than controls (β=‐0.15, *p* = 0.004) and there was a non‐significant trend towards smaller frontal gray matter volume (β=‐1.66, *p* = 0.056). Among females, but not males, TBI was significantly associated with smaller hippocampal (β=‐0.25, *p* < 0.0001), frontal gray matter (β=‐2.67, *p* =  0.007), and total cerebral gray matter (β=‐5.20, *p* =  0.030) volumes.

**Conclusion:**

In a community‐based cohort of older adults, TBI was associated with reductions in MRI hippocampal, frontal gray matter, and total cerebral gray matter volumes in females, but not males. Sex may portend different risk for later life atrophy after TBI, particularly in brain regions commonly affected by Alzheimer's disease and related dementias. Future work will evaluate TBI associations with longitudinal MRI trajectories.

